# Chirality and frequency measurement of longitudinal rolling of human sperm using optical trap

**DOI:** 10.3389/fbioe.2022.1028857

**Published:** 2022-12-12

**Authors:** Zhensheng Zhong, Can Zhang, Rui Liu, Jun He, Han Yang, Zijie Cheng, Tao Wang, Meng Shao, Shu Fang, Shengzhao Zhang, Hui Shi, Rufeng Xue, Huijuan Zou, Zeyu Ke, Zhiguo Zhang, Jinhua Zhou

**Affiliations:** ^1^ School of Biomedical Engineering, Anhui Provincial Institute of Translational Medicine, Anhui Medical University, Hefei, China; ^2^ Reproductive Medicine Center, Department of Obstetrics and Gynecology, The First Affiliated Hospital of Anhui Medical University, Hefei, China; ^3^ School of Science & Technology City, University of London, London, United Kingdom

**Keywords:** optical tweezers, sperm, longitudinal rolling, rolling chirality, rolling frequency

## Abstract

Motility is one of the most critical features to evaluate sperm quality. As longitudinal rolling of human sperm has long been ignored until recently, its detailed dynamics and cellular biological mechanisms are still largely unknown. Here we report an optical-tweezers-based method to evaluate the chirality and frequency of sperm rotation. According to the intensity distribution patterns of off-focus micron-size particles, we established a method to judge the orientation of the sperm head along the optical axis in the optical trap. Together with the rotation direction of the projection of the sperm head, the chirality of longitudinal rolling of sperm can be measured without the application of three-dimensional tracking techniques or complex optical design. By video tracking optically trapped sperm cells from different patients, both rolling chirality and rolling frequency were analyzed. In this study, all the vertically trapped human sperm cells adopt a right-hand longitudinal rolling. The orientation and rolling frequency but not the rolling chirality of sperm in the optical trap are affected by the trap height. The rotation analysis method developed in this study may have clinical potential for sperm quality evaluation.

## 1 Introduction

Infertility is a global health issue, which affects about 1/6 of couples worldwide (Sharlip et al., 2002). About 2.5%–12% of male population are infertile (Agarwal et al., 2015). As a result, evaluation of sperm quality plays important roles in both diagnosis of infertility and assisted reproductive technology. When sperm pass through the female reproductive tract, both the cervical mucus and the uterotubal junction served as barriers to filter out sperm with poor motility. Therefore, motility is considered as one of the most critical parameters to quantify the sperm quality, and poor motility is a common symptom of male infertility.

As sperm swims in three dimensions with relatively large velocity, it is a challenge to image the details of sperm moving trajectory. Currently in most sperm motility studies, conventional microscopy techniques, including bright-field, dark-field, and epifluorescence microscopy, have been applied to provide 2D or semi-quantitative 3D information (Bukatin et al., 2015; Hansen et al., 2019). Holographic microscopy and lens-free imaging technique allow quantitative three-dimensional tracking of sperm swimming (Su et al., 2012; Su et al., 2013; Gong et al., 2021). However, these techniques require precise interferometric optical path and the quality of the reconstructed 3D trajectories of swimming sperm depends on the imaging processing algorithm applied. Moreover, clinical sperm sorting technique require viscous solvent such as polyvinylpyrrolidone to reduce the swimming velocity of sperm. Nevertheless, in solutions with high viscosity or viscoelasticity, the rolling probability of bull sperm head is suppressed and the probability of asymmetry flagellar beating is increased, suggesting that the head rolling is potentially involved in sensation to different rheological properties in the female reproductive tract (Zaferani et al., 2021). These two problems can be solved by optical tweezers technique, which has been wildly applied in manipulation of microorganisms and particles (Kotsifaki et al., 2007; Kotsifaki et al., 2013; Serafetinides et al., 2017; Armstrong et al., 2020). By using optical tweezers, the swimming trajectory of a sperm is localized in a small region in the focal plane, which enables long-time recoding of sperm rotations with high temporal resolution without using viscous or viscoelastic solvents (Chow et al., 2017; Schiffer et al., 2020).

Early optical-tweezers based studies of sperm motility mainly focus on the power and force loaded onto the trapped sperm when it escapes the optical trap (Tadir et al., 1989; Tadir et al., 1990; Araujo et al., 1994; Dantas et al., 1995; Konig et al., 1996). By combining optical tweezers and fluorescent microscopy techniques, Nishimura *et al* revealed that the active digestion of sperm mitochondrial DNA is a two-step process: the mitochondrial nucleoid numbers are decreased gradually during spermatogenesis, and they are eliminated rapidly immediately after fertilization. (Nishimura et al., 2006). Previous studies demonstrated that the pre-trapping and post-trapping swimming speeds of a sperm does not change apparently, so that optical trapping enables quantitatively evaluation of sperm motility (Tadir et al., 1989; Nascimento et al., 2006). Since 2006, several optical trapping techniques were applied to achieve automatic and high-throughput sperm motility analysis (Nascimento et al., 2006; Shao et al., 2007a; Shao et al., 2007b; Nascimento et al., 2008; Shi et al., 2008; Dasgupta et al., 2010). Using optical tweezers, Hyun *et al* analyzed the effects of viscosity on sperm motility, discovered that with the increasing of solution viscosity, the curvilinear velocity of sperm decreases but the minimum laser power for trapping the sperm increases (Hyun et al., 2012).

Despite the great achievement of sperm motility studies using optical tweezers, investigation on rotation of optically trapped sperm was not reported until recently. Chow et al unraveled that the traces of sperm head in the optical trap is a ‘rose curve’ like trajectory in the focal plane (Chow et al., 2017), and Schiffer et al discovered that the head of optically trapped human sperm displayed longitudinal rolling with full 360° rotations at a frequency of about 4–8 Hz, no matter it is CatSper-deficient or not (Schiffer et al., 2020). Nevertheless, without characterizing the orientation of the sperm in the optical trap, these studies did not focus on the longitudinal rolling chirality of sperm.

**FIGURE 1:**
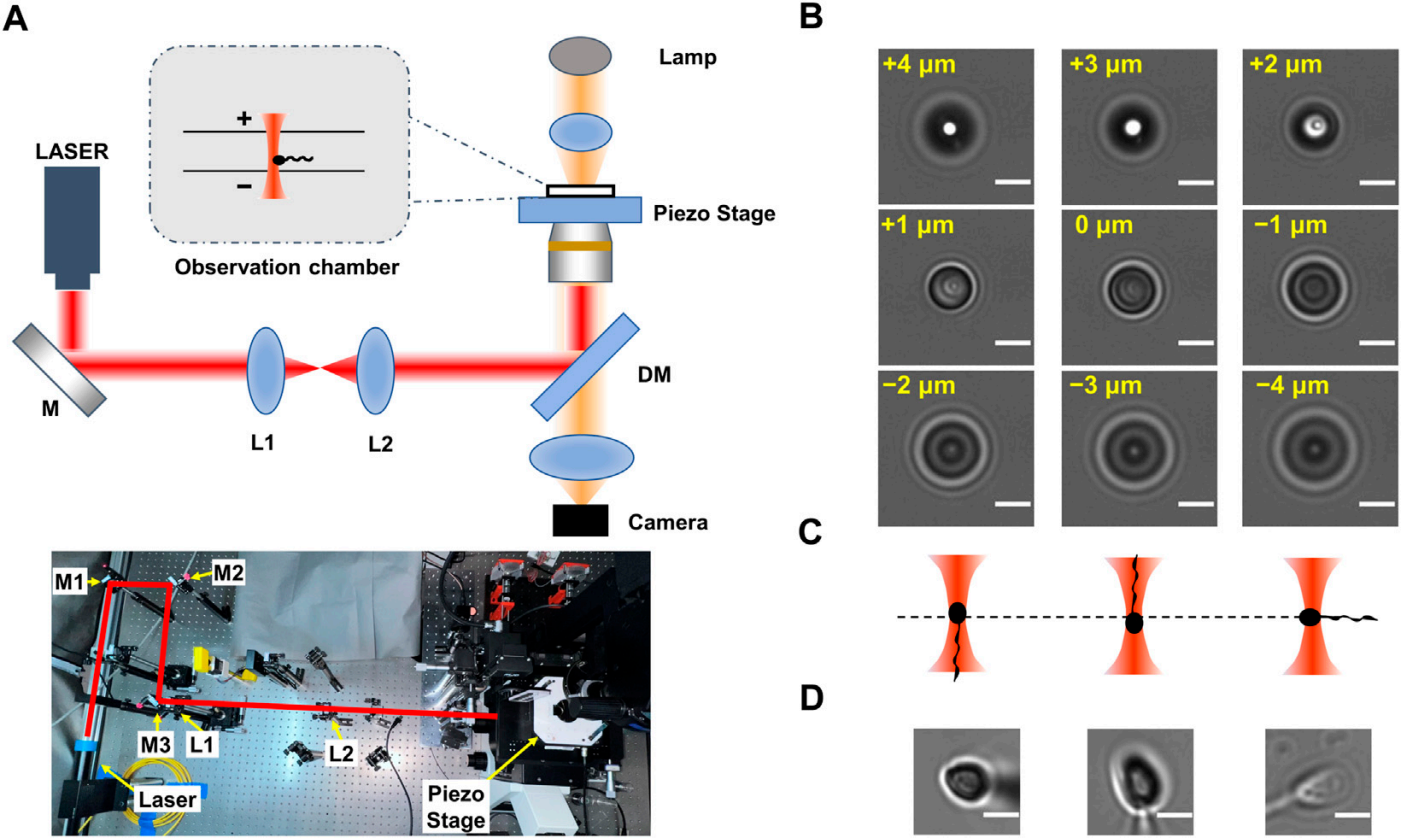
Principle of determination of the flagellar orientation of optically trapped sperms. **(A)** Experimental setup. The upper panel is the scheme of experimental setup. Here ‘+’ indicates the orientation that is away from the objective lens and ‘-’ indicates the orientation that is towards the objective lens. The lower panel is the photograph of the optical tweezers device. The optical path is indicated by the red lines. **(B)** Series of images of a 3 μm polystyrene bead stuck at the glass surface. The displacement of the microscope stage was labeled in yellow. **(C)** Scheme of optically trapped sperm with downward, upward, and horizontal flagellum, respectively. **(D)** The typical images of optically trapped sperms. The left, central and right panels showed optically trapped sperms with downward, upward, and horizontal flagellum, respectively. The scale bar of each graph is 3 μm in length.

In this study, we established a method to analyze the longitudinal rolling dynamics of single sperm based on optical trapping technique (Figure 1). The chirality of longitudinal rolling was determined by recognizing both the rolling direction of the projection of the optically trapped sperm head and the orientation of the head along the optical axis. We also analyzed the rolling frequency of sperm cells of different rolling direction. This method may simplify the sperm rolling research, and may have potential clinical values on single-sperm quality examination, which is crucial in intracytoplasmic sperm injection (ICSI) treatment.

## 2 Materials and methods

### 2.1 Sample preparation

The human semen samples were collected from five individual patients seeking *in vitro* fertilization treatment at the Reproductive Medicine Center, the First Affiliated Hospital of Anhui Medical University. All patients gave their informed consents prior to their inclusion in this study. The semen sample was diluted in fertilization medium (Cook Medical, United States) and stored in 4°C before the optical trapping experiment in the same day. The sperm samples were further diluted in 10 to 50 folds using fertilization medium before loading on the observation chamber of the home-built optical tweezers instrument (Figure 1A).

### 2.2 Single-sperm manipulation using optical tweezers

The single-sperm manipulation experiments were performed in a temperature (25 ± 1 °C) controlled room. The optical trap was generated using a fiber laser with a power of ∼400 mW (Amonics Ltd. AFL-1064–37-R-CL, Hong Kong, China) (Figure 1A). The laser beam was expanded by a telescope setup of two lenses (L_1_ and L_2_ in Figure 1A), and focused by a water immersion objective (×60, NA 1.20, Olympus, Japan). The sperm samples of Patient A, B and C were loaded on a coverslip-made chamber with internal height of ∼280 μm. To explore the effect when the optical trap is close to upper surface of the chamber, a coverslip-made chamber with internal height of ∼140 μm was also used. The optical trap was 10–80 μm above the lower surface of the chamber. Each sperm was trapped for no longer than 20 s. The chamber position was adjusted by a servo-controlled, three-dimensional piezoelectric stage (P-563.3CD, Physik Instrumente, Karlsruhe, Germany). The videos of sperm rotation were recorded by a CMOS camera (MV-SUA231GM-T, MindVision, China) at a frequency of 90 frame per second (for samples from Patient B and C) or 200 frame per second (for samples from Patient A). For each video, a region of interesting (ROI) with a typical size of 90 × 90 pixel was selected using ImageJ, and the rolling direction and rolling frequency of the projection of each sperm head was determined *via* manually frame-by-frame analysis.

## 3 Results

### 3.1 Determination of longitudinal rolling charity of sperm head in the optical trap

As the camera records only 2-dimension trajectory of the 3-dimension movement of sperm, to determine the longitudinal rolling charity of sperm, both the relative location between head and tail, as well as the rotation direction of the projection of the sperm head on the camera, should be recognized. The lengths of the head, midpiece and tail of a typical sperm cell are 4–6 μm, 5–7 μm and 50–60 μm, respectively (Cummins and Woodall, 1985), while the focal plane is about 1 μm in depth. If a motile sperm is vertically trapped, most part of its tail should be off-focus. As the fraction of sperm tail adjacent to the midpiece is slightly off-focus, the orientation of sperm tail along the optical axis can be determined by observation of the diffraction pattern of its projection.

By imaging a 3 μm polystyrene bead (ThermoFisher) stuck in the glass surface at difference focal depths (Figure 1B), we determined the relation between the diffraction pattern and the off-focus distance of a micron-size transparent particle. When the particle is 1–4 μm above the focal plane (i.e., it is 1–4 μm more far away from the objective lens than when it is within the focal plane), its central area is brighter than that when the bead center is in the focal plane. Inversely, when the trapped object is 1–4 μm below the focal plane (i.e., it is 1–4 μm closer to the objective lens), its central area is darker.

**FIGURE 2:**
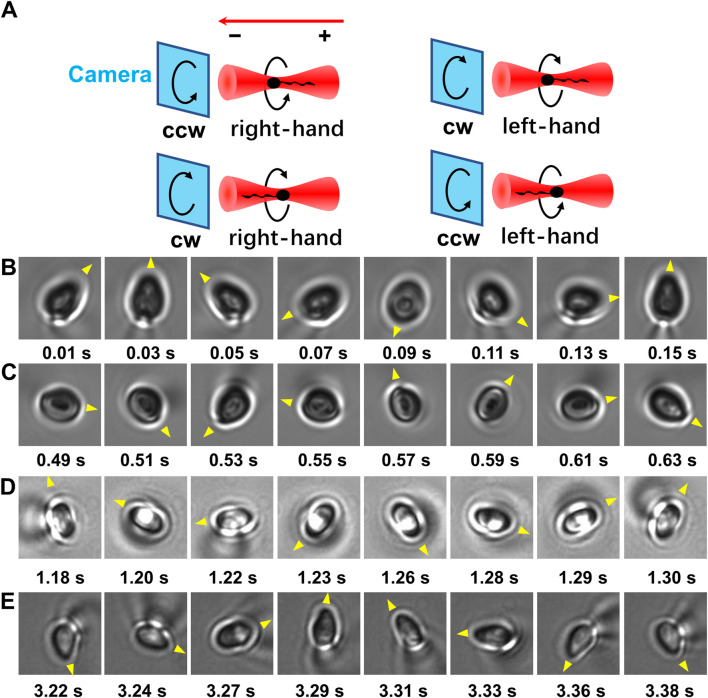
The longitudinal rolling chirality of vertically trapped sperm. **(A)** Scheme of vertically trapped sperm with left-hand and right-hand longitudinal rolling chirality. The relations are showed among the orientation of vertically trapped sperm, the rotation direction of the sperm head projection, and the longitudinal rolling chirality of the sperm. Here the red arrow indicates the propagation direction of illimitation light, ‘+’ indicates the orientation of the sperm tail that is away from the objective lens, and ‘−’ indicates the orientation that is towards the objective lens. Noticed that the images of the rolling sperm are recorded by the camera in bottom view. **(B)** A right-handed rolling sperm from Patient A with an upward tail and a counter-clockwise rolling head projection. **(C)** A right-handed rolling sperm from Patient A with a downward tail and a clockwise rolling head projection. **(D)** A right-handed rolling sperm from Patient B with an upward tail and a counter-clockwise rolling head projection. **(E)** A right-handed rolling sperm from Patient C with an upward tail and a counter-clockwise rolling head projection. The yellow triangles indicate the rotation direction of the projection of each sperm head.

As the centroid of head of a trapped sperm cell is in the focal plane, whether the sperm tail is above or below the focal plane can be determined according to the relation between the diffraction pattern and the off-focus distance of micron-size object. Once the relative position between the head and tail of the trapped sperm is determined, the orientation of the sperm cell in the optical trap can be determined. Specifically, if the tail of a vertically trapped sperm is downward, it should be beneath the focal plane (i.e., closer to the objective lens) (Figure 1C, left panel). In this situation, the axial part of the tail projection is darker than its edge part as well as the center of projection of the sperm head (Figure 1D, left panel). Inversely, when the sperm tail is downward, it should be above the focal plane (Figure 1C, middle panel). In this case, the axial part of the tail projection is brighter than its edge part as well as the center of projection of the sperm head (Figure 1D, middle panel). By contrast, if the sperm is horizontally trapped (Figure 1C, right panel), the intensity distribution pattern of the tail projection is close to that of the sperm head (Figure 1D, right panel). As a result, by comparing the diffraction pattern of the sperm head with that of the tail, the orientation of the sperm head along *z* axis can be determined. Combining with the rotation direction of the projection of the sperm head from the video, the chirality of the longitudinal rolling of vertically trapped sperm can be further recognized (Figure 2 and Supplementary Videos S1, S2). In detail, a vertically trapped sperm rolls right-handedly along the longitudinal axis, if its tail is upward and the projection of the head rotates counterclockwise, or its tail is downward and the projection of the head rotates clockwise (Figure 2A, upper left and lower left panels). In contrast, a vertically trapped sperm rolls left-handedly if its tail is upward and the projection of the head rotates clockwise, or its tail is downward and the projection of the head rotates counterclockwise (Figure 2A, upper right and lower right panels). Interestingly, we also recorded motion of a morphologically abnormal sperm in previous study, whose trapping position was the midpiece (Supplementary Video S3), and its projection is obviously different from those of morphologically normal spern cells in the trap.

### 3.2 Effect of optical trap height on the longitudinal rolling of human sperm

As the full length of normal human sperm is 60–70 μm, the height of the optical trap, i.e., the distance between the optical trap center and the lower surface of the chamber, may affect the orientation and rolling behavior of the trapped sperm. To evaluate this effect, 11 sperm cells donated by Patient A were arrested by optical trap when the trap height is 10 μm, and the rolling movement of each sperm was recorded by the camera. Then, the optical trap height was adjusted by controlling the piezo stage, and the rolling movement of the same sperm was also recorded when the trap height was increased to 30, 50, and 80 μm, respectively (Table 1). When the trap height is 10 μm, 9 sperm cells are horizontally oriented and two sperm cells are with upward tail, and no obvious pausing observed during the rolling movement of the sperms. With the trap height increasing, the ratio of horizontally oriented sperm decreased and ratio of sperm with downward tail increased. Moreover, the ratio of sperm with pausing and stuck events also increased. Here, ‘pausing’ is defined as the longitudinal rolling stops but it restarts later, and ‘stuck’ is defined as the longitudinal rolling stops and did not restart during the observation. Worth mentioning, those stuck sperm cells without obvious longitudinal rolling exhibit swinging-like movement (Supplementary Video S4). With the trap height increasing, the averaged rolling frequency of the vertically trapped sperm cells decreased. However, when the sperm cells with pausing and stuck events are excluded, the averaged rolling frequency of the vertically trapped sperm cells did not change significantly with different trap heights (Table 1).

**TABLE 1:** Summary of rotation and orientation of optically trapped sperm cells with different trap heights.

	Trap height (μm)	Tail orientation			Number of sperm cells with pausing or stuck behavior^a^	Head rolling frequency (Hz) (mean ± s.e.m.)	Head rolling frequency^a^ (Hz) (mean ± s.e.m.)
		Horizontal	Upward	Downward			
A	10	9	2	0	0	7.7 ± 1.1	7.7 ± 1.1
	30	7	4	0	6	4.0 ± 1.2	8.0 ± 0.4
	50	1	4	6	4	5.0 ± 0.9	6.5 ± 0.8
	80	0	2	9	9	2.0 ± 0.7	NS^b^
B	10	82	18	0	11	8.0 ± 0.4	8.7 ± 0.4
C	10	80	10	0	2	7.4 ± 0.3	7.5 ± 0.3
D	120^c^	1	1	28	1	8.6 ± 0.6	8.6 ± 0.6
E	120^c^	1	1	28	1	5.0 ± 0.4	5.1 ± 0.4

^a^ Sperm cells with pausing or stuck behavior are excluded.

^b^ Sample size is too small to be statistically significant.

^c^ The optical trap is 20 μm below the upper surface of the chamber.

### 3.3 Human sperm from each patient rolls right-handedly

Interestingly, sperm cells with upward tail exhibited counterclockwise rotated projection of the head (Figure 2B), and sperm cells with downward tail exhibited clockwise rotated projection of the head (Figure 2C), i.e., all the vertical trapped sperm cells from Patient A adopted right-handed longitudinal rolling.

To further confirm the longitudinal rolling charity of human sperm, 190 motile sperm cells from two more patients were trapped and analyzed using optical tweezers with the trap height of 10 μm (Table 1). Briefly, 162 and 28 sperm cells were trapped with horizontal and upward tail, respectively. No sperm cells with downward tail were observed. For all sperm cells from both patient B and C, all those sperm cells with upward tail exhibited counterclockwise rotated projection of the head (Figures 2D,E), i.e., all the vertical trapped sperm cells adopted right-handed longitudinal rolling, which are the same as those from Patient A.

To explore the effect when the optical trap is close to upper surface of the chamber, we performed the optical trapping experiment of sperm cells from two more patients (D and E) in a thinner chamber (internal height ∼140 μm), and the trap height was set to 120 μm, i.e. ∼20 μm away from the upper surface (Table 1). Interestingly, in this experimental condition, for each patient, 1, 1 and 28 sperm cells are with horizontal, upward and downward tail, respectively. All the vertical trapped sperm cells from Patient D and E adopted right-handed longitudinal rolling, further indicating the right-handed chirality of longitudinal rolling of human sperm. The asymmetry of the optical force along the *z* axis may be the major factor leading to the less horizontally oriented sperm cells when the trap is close to the upper surface than to the lower surface, as the maximum restoring force along the negative half of *z* axis is larger than along the positive half of *z*-axis (Zhou et al., 2012).

The head rolling frequencies of optically trapped sperm cells with different orientation were also summarized in Table 2. The head rolling frequencies of trapped sperm cells with horizontal, upward and downward tail are 8.3 ± 0.3, 6.7 ± 0.6 and 6.9 ± 0.4 Hz, respectively. The frequencies we observed are in consistent with previous reported values of 4–8 Hz (Schiffer et al., 2020).

**TABLE 2:** Head rolling frequencies of optically trapped sperm cells with different orientation.

Tail orientation	Number of sperm cells^a^	Head rolling frequency^a^ (Hz) (mean ± s.e.m.)
horizontal	163	8.3 ± 0.3
upward	37	6.7 ± 0.6
downward	60	6.9 ± 0.4

^a^ Sperm cells with pausing or stuck behavior are excluded.

## 4 Discussion

When the sperm head is deviated from the trap center, a torque on the sperm head around the trap center is exerted by the optical field. The sperm head in an optical trap can be considered as a triaxial ellipsoid, and the torque exerted by the optical field automatically regulates the orientation of the ellipsoid to its longest axis parallel to the optic axis, according to our previous theoretical calculations (Zhou et al., 2012; Shao et al., 2019). Because the longest axis of sperm head is parallel to the longitudinal axis of the sperm, sperm cells tend to be vertically trapped. Another torque generated by the beating of sperm tail leads to the longitudinal rolling of the sperm head. These two torques are not in balance, resulting in the rolling of sperm head in the optical trap.

In this study, we unraveled the effect of optical trap height on the rolling behavior of the trapped sperm. Our study indicates that, when the optical trap is close to the lower surface of the chamber, the tail of the trapped sperm is compelled to adopt horizontal or upward orientation, resulting in less interaction with the surface of the chamber. With trap height increasing, the probability of the sperm tail interacts with the surface increases and the pausing and stuck events increase. Noticed that although the normal sperm cell is 70 μm in length, the sperm cells we investigated in the experiment are donated from patients asking for IVF treatment. It is possible that the tail of the trapped sperm is abnormally long, so it may interact with the lower surface of the chamber, even when the trap height is 80 μm above the lower surface. The uncertainty of the trap height may also contribute to the possibility of the interaction. We measured the axial shifting of the optical trap (∼1.6 μm/min) by recording the image of a bead stuck in the lower surface of the chamber. These results indicate that the interaction between the sperm tail and the surface of the glass chamber may bring tortional constraint to the sperm and pause the longitudinal rolling of sperm.

In this study, the frequencies of longitudinal rolling of optically trapped sperm were measured manually. The averaged rolling frequencies of sperm cells for five patients are close, and are consistent with previous reports (Smith et al., 2009; Subramani et al., 2014; Schiffer et al., 2020). Besides the longitudinal rolling, the optically trapped sperm may display other rotation motions, such as the precession of the rolling axis around the optical trap center, which increases the complexity of intensity pattern of the rolling sperm by algorithm. Interestingly, it is likely that the right-handed rolling human sperm prefers a left-handed presession (Supplementary Video S5), whose projection of the head on the camera is in accordance with the ‘rose curve’ like trajectory reported previously (Chow et al., 2017). To achieve automatic measurement of longitudinal rolling frequency in bright-field, physical parameters, such as the coordination of the sperm centroid may be considered in the future.

Previous studies have been reported that counterion and viscosity of medium may significantly affect the rolling behavior of human sperm. Chiffer et al. reported that the rotation frequency of human sperm increases with increasing of bicarbonate concentration, but not with increasing of calcium concentration (Schiffer et al., 2020). Viscosity as well as viscoelasticity of the medium also play complicated roles in the motion of sperm (Schiffer et al., 2020; Zaferani et al., 2021). In this study, to avoid uncertain effects on sperm motion as well as on the optical trap causing by the medium, a standard sperm dilution medium for *in vitro* fertilization (Cook Medical, United States) was used. In the future, using the method developed in this study, we will explore how chemical and physical properties of medium affect the motion and optical trapping of human sperm.

In current clinical practice of ICSI, the single sperm cell for injection is picked manually by trained technicians, which is laborious and the fertilization rate relies on the experience of the technician (Oseguera-Lopez et al., 2019). On the other hand, optical tweezers have been demonstrated as an efficient tool for spermatozoa isolation (Shao et al., 2007b; Auka et al., 2019). Combining the optical-tweezers based single-cell manipulation technique with the sperm rotation characterization method established in this study, automatic evaluation and isolation of single sperm may be achieved in the future, which may reduce the manual subjective error and increase the efficiency of sperm preparation during the ICSI procedures.

## 5 Conclusion

In summary, we established an optical-tweezers based method to determine the chirality of sperm rotation using optical tweezers, in which three-dimensional tracking technique is not required. Without using complex optical design or data analysis algorithm, the method we established in this study may simplify the research on sperm rotation. Our study indicates that human sperm prefers right-hand longitudinal rolling. The effect of optical trap height on the sperm longitudinal behavior was also investigated in this study.

## Data Availability

The original contributions presented in the study are included in the article/Supplementary Material, further inquiries can be directed to the corresponding authors.
